# The Mathematics Anxiety-Complexity Effect in Word Problems

**DOI:** 10.1177/17470218261425251

**Published:** 2026-02-17

**Authors:** Gabriella Daroczy, Felix Cramer, Christina Artemenko, Thomas E. Hunt, Hans-Christoph Nuerk

**Affiliations:** 1 Department of Psychology, University of Tuebingen, Germany; 2 LEAD Graduate School & Research Network, University of Tuebingen, Germany; 3 German Centre of Mental Health (DZPG), University of Tuebingen, Germany; 4 School of Science, University of Derby, UK

**Keywords:** mathematics anxiety, word problems, arithmetic complexity, lexical consistency, anxiety-complexity effect, online experiment

## Abstract

Word problems are notoriously difficult for students to solve and require both mathematical and linguistic processes. Moreover, word problem-solving performance might also be negatively affected by mathematics (math) anxiety, particularly when task complexity increases. Therefore, the study investigated whether math anxiety impacts word problem performance, particularly when mathematical difficulty (i.e., arithmetic carry/borrow operations) or linguistic difficulty (i.e., lexical consistency) increases. In an online experiment with *N* = 129 adults, we observed that higher math anxiety levels were related to lower performance in word problem-solving. Moreover, math anxiety particularly affected performance in linguistically – but not arithmetically – difficult word problems. This suggests that math-anxious individuals particularly struggle during word problem-solving when the text was more difficult to translate into an arithmetic problem. Therefore, the anxiety-complexity effect in word problems holds for math anxiety affecting the resolution of linguistic complexity rather than arithmetic complexity.

## Introduction

Mathematics anxiety is a well-established phenomenon characterized by a feeling of tension or fear experienced by individuals when solving mathematical problems (Dowker et al., 2016). Math-anxious individuals already face many challenges in mathematics, but word problems can be difficult for many individuals (e.g., Verschaffel et al., 2020). This presents a unique challenge that warrants further investigation. It has been established that both arithmetic and linguistic processes can contribute to word problem-solving performance in adults (Daroczy et al., 2020; see Daroczy et al., 2015, for a review). However, the influence of emotional processes on word problem-solving is much less clear. Math anxiety has been found to impair arithmetic processes involved in solving a math problem (e.g., Skagerlund et al., 2019). However, it is unclear whether math anxiety also indirectly impairs other processes, such as linguistic processes, involved in mathematical problem-solving.

Math anxiety can have a differential impact on performance across different types of word problems (e.g., Novak & Tassell, 1997). A limited number of studies have separately examined the influence of arithmetic and linguistic complexity in word problems. In this context, mathematical difficulty may be seen as encompassing the entire math problem, while arithmetic difficulty focuses on the specific arithmetic operations, such as the carry and borrow operations. Linguistic difficulty pertains to the linguistic aspects, such as nominalization. The question arises whether math anxiety impacts not only word problem-solving performance and arithmetic processes but also the resolution of linguistic complexity. Because both models on word problem-solving and models on math anxiety assume shared processes, the current study aims to explore the influence of math anxiety on word problem performance and the extent to which its impact varies not only with arithmetic difficulty (i.e., inclusion of a carry/borrow operation) but also with linguistic difficulty (i.e., lexical consistency).

### The Impact of Mathematics Anxiety on Math Performance

Literature consistently shows a negative association between math anxiety and math performance (*r* *≈* −.30; for meta-analyses, see Barroso et al., 2021; Zhang et al., 2019). The deficit theory suggests that poor performance leads to higher anxiety, while the debilitating anxiety model suggests that anxiety impairs performance (Carey et al., 2016). These considerations highlight a bidirectional relationship between mathematics anxiety and performance. According to the attentional control theory (Eysenck et al., 2007), the anxiety-induced performance deficit is caused by reduced processing efficiency due to impaired attentional control. Attention is regulated both by a goal-directed attentional system and a stimulus-driven attentional system. While task performance is typically guided by goal-directed attention, anxiety makes task-irrelevant stimuli such as worrying thoughts more salient, thereby enhancing stimulus-driven attention and distractibility. Hence, anxiety disrupts the balance between the two systems, leading to reduced processing efficiency. Supporting the attentional control theory, previous research has shown that individuals with higher working memory capacity, in particular, are impaired in their performance when they experience math anxiety (Cuder et al., 2023; Ramirez et al., 2013, 2016). This might be explained by math anxiety hindering the use of advanced, memory-demanding strategies that these individuals typically employ (Ramirez et al., 2013, 2016). Because such strategies are particularly required for more complex tasks, math anxiety should affect performance more in difficult compared to simple math tasks.

Indeed, such an anxiety-complexity effect has been repeatedly shown, indicating that math anxiety affects performance in complex arithmetic more than in simple arithmetic (Ashcraft & Faust, 1994; Ashcraft & Krause, 2007; Faust et al., 1996; Huber & Artemenko, 2021). The most prominent explanation is that math anxiety preoccupies working memory so that fewer resources are available to solve complex arithmetic tasks which necessitate working memory, leading to worse performance (Ashcraft & Faust, 1994). As solving word problems requires working memory as well (e.g., Andersson, 2007), the attentional control theory would predict that math anxiety affects performance in word problems as well. The negative relation between math anxiety and performance was indeed shown for word problems (Barroso et al., 2021; Novak & Tassell, 2017; van Dijck et al., 2022): Students with high levels of math anxiety tend to perform poorly on such word problems compared to those with low levels of math anxiety.

Word problems are complex math tasks, naturally calling for a diverse set of skills (Strohmaier et al., 2022), and are prone to anxiety (Khoshaim, 2020). For example, Lai et al. (2015) presented participants with word problems ranging from easy to difficult and found that math anxiety had a negative impact on problem-solving performance. Importantly, math anxiety influences performance even in simple arithmetic word problems (e.g., Vukovic et al., 2013). Furthermore, besides actual difficulty, perceived difficulty of word problems mediates the math anxiety-performance link (Doz et al., 2023).

Arithmetically difficult and linguistically complex word problems may be particularly susceptible to the effects of math anxiety. The question remains whether math anxiety influences only the arithmetic aspects of the word problem or also the linguistic aspects. Thus, we will investigate whether the impact of math anxiety on performance in word problems is predominantly shaped by arithmetic difficulty or by linguistic complexity.

### The Influence of Arithmetic and Linguistic Difficulty in Word Problems

Arithmetic difficulty might play a role in the relation between math anxiety and word problem-solving. For example, the anxiety-complexity effect was found for carry and borrow operations (e.g., Faust et al., 1996; Huber & Artemenko, 2021), and the question is whether it also generalizes to word problems. The carry operation in two-digit addition means that a decade needs to be carried from the unit to the decade position if the sum of the units is larger than 9 (e.g., 48 + 17 vs. 42 + 23). Similarly, the borrow operation in two-digit subtraction means that a decade needs to be borrowed if the unit of the minuend is smaller than the unit of the subtrahend (e.g., 65–17 vs. 65–23). Thus, both the carry operation in two-digit addition and the borrow operation in two-digit subtraction increase the difficulty of arithmetic (e.g., Artemenko et al., 2018) and word problems (Daroczy et al., 2020). The role of the carry/borrow operation is especially important, given that it necessitates working memory (for reviews, see DeStefano & LeFevre, 2004; Imbo et al., 2007). Emphasizing the relevance of working memory in the context of math anxiety effects, several studies have shown that math anxiety influences performance on multidigit arithmetic problems involving the carry/borrow operation, constituting the anxiety-complexity effect (Ashcraft, 2002; Ashcraft & Kirk, 2001; Chan & Tang, 2020; Faust et al., 1996; Huber & Artemenko, 2021; Hunt & Sandhu, 2017; Hunt et al., 2014). Thus, we expect that math anxiety will have a larger impact on performance for more difficult word problems requiring carrying or borrowing, generalizing the anxiety-complexity effect from arithmetic to word problems.

Carry and borrow operations occur in calculations with addition and subtraction, respectively. The type of arithmetic operation, however, also influences performance in word problems, with subtraction found to be more difficult than addition (Daroczy et al., 2020). A differential influence of math anxiety on different operations has not yet been found for arithmetic (Huber & Artemenko, 2021); however, the (missing) anxiety-complexity effect for arithmetic operations has not yet been studied in word problems, which pose additional demands to the individual. Therefore, by testing the influence of math anxiety on the carry/borrow and operation effects, we can either generalize previous findings on the anxiety-complexity effect to word problems or discover differences due to the higher task complexity when arithmetic problems are embedded in texts. In sum, carry/borrow and operation effects increase difficulty in word problems, which might be further influenced by math anxiety.

Besides arithmetic skills, the key aspect of word problems is that they also demand linguistic skills. The difficulty of word problems depends on multiple linguistic factors (for a review, see Daroczy et al., 2015). For instance, performance in word problems is affected by complex or ambiguous words (Cummins et al., 1988; De Corte et al., 1985; Shaftel et al., 2006), less frequent words, and complex syntax (Plath & Leiss, 2018). One of the most researched linguistic factors is lexical consistency. Misleading lexical inconsistency can increase complexity through cue words that signal or imply a particular operation (e.g., “Peter lost money” signals a subtraction), when in fact another operation is required (Hinsley et al., 1977; Lewis & Mayer, 1987). Lexically inconsistent wording causes slower and less accurate responses than lexically consistent wording (Daroczy et al., 2020; Hegarty et al., 1992, 1995). As math anxiety influences the reading pattern in complex word problems (Strohmaier et al., 2022), it might also have a differential impact on word problems with different linguistic complexities. Namely, the lexical consistency effect could be enlarged due to math anxiety, because lexically inconsistent problems are more complex and presumably require more working memory (see Daroczy et al., 2015, for a review), and working memory plays a crucial role between math anxiety and performance (according to the attentional control theory; Eysenck et al., 2007). However, this has not been investigated yet. In case the anxiety-complexity effect also holds for linguistic difficulty, a larger consistency effect would be expected for individuals with higher levels of math anxiety.

Math anxiety might not separately impact arithmetic and linguistic difficulty. Rather, the impact of math anxiety might be more severe for word problems that are both mathematically and linguistically complex. Such problems require individuals to understand complex mathematical concepts while also deciphering the language used in the problem. Although linguistic and arithmetic complexity are functionally entangled in word problems, their interaction is rarely explored (De Corte et al., 1990; Daroczy et al., 2020). Nevertheless, Daroczy et al. (2020) observed interactions between lexical consistency and operation type (existence of a carry operation). Such interactions increase the difficulty of the word problem so much that the cognitive load in working memory might be increased to the point of impairing the whole word problem solution process even more than predicted by the additive difficulty of linguistic and arithmetic complexity. As math anxiety is considered to occupy working memory resources (according to Attentional Control Theory [ACT]; Eysenck et al., 2007), which are especially needed when a problem is both arithmetically and linguistically complex, individuals with higher levels of math anxiety may struggle with these types of problems. Importantly, this would suggest that the impact of math anxiety on math performance would be more than additive when arithmetic and linguistic complexity come together.

### Objectives

The present web-based experiment was preregistered at: https://aspredicted.org/xd56e.pdf. This study investigates if math anxiety differentially impairs performance on word problems depending on arithmetic and linguistic complexity and their interaction. First, as a validity check, thereby replicating previous studies, increased arithmetic complexity (operation effect and carry/borrow effect) and linguistic complexity (consistency effect) are expected to reduce word problem performance independently and via an interaction. Therefore, the factors will be manipulated independently of one another (i.e., orthogonally). Second, word problem performance is expected to be negatively impacted by higher math anxiety. Specifically, due to the anxiety-complexity effect, which is expected to generalize beyond arithmetic complexity, we anticipate this impairment for both arithmetic and linguistic complexity. Finally, the influence of math anxiety is expected to be over-additively stronger when arithmetic and linguistic factors are both complex.

## Method

### Participants

Deviating from the preregistration, data were only collected for the German sample.^1^ The final sample consisted of *N* = 129 adults (101 females and 24 males, age (years): *M* = 23.50, *SD* = 5.05, *Range* = 18–56).^2^ Out of 780 clicks on the link, 386 individuals started the study. As preregistered, participants were excluded due to non-completion (*n* = 226),^3^ not being native in German (*n* = 5), survey completion on tablet/smartphone (*n* = 0), very or extremely noisy environment during study completion (*n* = 3), responding dishonestly (*n* = 7), accuracy below 50% (*n* = 16), and less than 80% of Abbreviated Math Anxiety Scale (AMAS) completed (*n* = 0).

Participants gave their consent and received either course credits or participation in a lottery for vouchers. The web-based study was in accordance with the latest version of the Declaration of Helsinki and was approved by the Ethics Committee for Psychological Research of the University of Tuebingen.

### Materials

#### Word Problems

Three within-subject factors were tested through word problems (see Supplementary Material, Table S1): the arithmetic factors carry/borrow (with/without) and operation (addition/subtraction), and the linguistic factor lexical consistency (lexically consistent/inconsistent). In this 2 × 2 × 2 design, each of the resulting eight conditions occurred in six word problems, for a total of 48 word problems. The word problems were one-step calculation problems chosen as a subset from our previous study (Daroczy et al., 2020) only including the verbalized form. Lexical consistency was manipulated by systematically varying both the keyword and the required arithmetic operation: The second or third sentence contained a keyword, which evoked an operation (e.g., “sell”/“selling” for subtraction, “buy”/“buying” for addition). The following example shows a lexically inconsistent word problem which requires a subtraction, including a borrow operation, to solve:

S1: Ein Dieb hat einer Frau einige Diamanten gestohlen.

Translation: *A thief stole some diamonds from a woman.*

S2: Der Dieb hatte ihr 54 Diamanten gestohlen.

Translation: *The thief had stolen 54 diamonds from her.*

S3: Die Polizei findet 28 Diamanten wieder.

Translation: *The police recover 28 diamonds.*

S4: Wie viele Diamanten muss die Polizei noch finden?

Translation: *How many diamonds are left for the police to find?*

To keep the word problems solvable and minimize the burden on working memory, no unnecessary information was included. All word problems belonged to the “change” type as categorized by Riley et al. (1983). A change word problem is a type of mathematical word problem that involves determining how much an amount or quantity has changed over time or because of certain actions or operations. We also ensured that each task could be clearly solved with one calculation, that the text was not ambiguous, and that there was only one correct solution. Finally, all sentences were constructed in active verb forms, because the active voice helps people to understand word problems better (Abedi et al., 2005; Shaftel et al., 2006).

All problems included two two-digit numbers in Arabic notation as operands, involving either addition or subtraction as the arithmetic operation. For numerical difficulty, half of all subtraction problems required a borrow to solve, and half of the addition problems required a carry. Overall problem size and parity were matched between the conditions. Pure decades (e.g., 50), tie numbers (e.g., 66), unit ties (e.g., 23–13), decade ties (e.g., 41 + 43), and mirror numbers (e.g., 24–42) were excluded to prevent any automatic mental retrieval. The word problem, however, was constructed such that both operations, addition and subtraction, were equally likely for each keyword type, resulting in 50% lexically consistent and 50% lexically inconsistent trials. A lexically consistent word problem without a carry or borrow was the easiest type of problem used in the present study; conversely, a lexically inconsistent word problem calling for a carry or borrow reflects an over-additive difficulty and is considered the most challenging (see Supplementary Material, Table S1).

#### Cognitive Skills

*Arithmetic skill* was assessed by the subtests for addition and subtraction of a speeded measure of arithmetic skills, similar to the Math4Speed (Loenneker et al., 2024). Both subtests contained 28 arithmetic problems and no text. All problems comprised two-digit numbers (number pairs were different from those used for word problems) with and without a carry/borrow operation, in a fixed randomized order. The time limit for each subtest was 90 s. As a measure of arithmetic skill, we used the mean number of correctly solved items from the two subtests (scale of 0–28) in this study. The internal consistency was very high (α = .94, which was estimated using the Spearman–Brown formula from the correlation between the addition and subtraction subscores).

*Reading comprehension* was measured by presenting a reading comprehension test in German comprising 56 items. Each item contained a short statement (e.g., “horses have hooves” or “rain is dry”) which had to be judged as “right” or “wrong” by participants. For every correctly answered item, one point was assigned. Thus, the overall sum, ranging from 0 to 53, indicates reading comprehension skill, with higher scores indicating better reading comprehension. The test was developed based on the sentence verification task by Collins and Quillian (1969). The time limit for the test was 90 s. The reliability was very high in our sample, as indicated by the Kuder–Richardson 20 coefficient (KR-20 = .96, which is the Cronbach’s α for binary items).

#### Math Anxiety

*Math anxiety* was assessed by the AMAS (Hopko et al., 2003) in the German translation (Huber & Artemenko, 2021; https://osf.io/3mdb2). Participants were asked to state on a 5-point Likert scale (1 = *low anxiety*, 5 = *high anxiety*) how anxious they would feel in each math situation described in the 9 items. Accordingly, higher values of the resulting sum scores (ranging from 9 to 45) indicate higher levels of math anxiety. The AMAS is an appropriate instrument for the assessment of math anxiety in web-based studies (Cipora et al., 2017). The internal consistency was very good in our sample (α = .92).

#### Other Anxiety Measures

Other forms of anxiety, such as general anxiety and test anxiety, can also have an impact on math performance (Dowker et al., 2016; Hembree, 1988; Ramirez & Beilock, 2011). Thus, these anxiety constructs may confound the relation between math anxiety and word-problem performance and should therefore be controlled for.

*State anxiety* was measured via the short state-version of the State and Trait Anxiety Inventory (Taylor & Deane, 2002) in German (Englert et al., 2011). Participants were asked to indicate their current state of anxiety on a 4-point Likert-scale (1 = *not at all*, 4 = *very*). The sum score of 5 items (overall scale ranging from 5 to 20) is considered to represent state anxiety, with a higher score indicating higher anxiety. The internal consistency was very good in our sample (α = .87).

*General anxiety* was assessed by the Generalized Anxiety Disorder 7 (GAD-7, Spitzer et al., 2006) in its German version (Löwe et al., 2008). The 7-item scale was developed for identifying likely cases of GAD based on clinical criteria (DSM-IV). Participants were asked to evaluate their anxiety symptoms of the last 2 weeks on a 4-point Likert scale (0 = *not at all*, 3 = *nearly daily*). The scale ranges from 0 to 21 points, with higher values indicating higher anxiety. With a threshold of 10, the GAD-7 assumes an excellent sensitivity of 89% and a specificity of 82% for GAD (Spitzer et al., 2006). The internal consistency was good in our sample (α = .84).

### Procedure

Data collection was web-based, implemented at pavlovia.org with jsPsych (de Leeuw et al., 2023). The link to the study was distributed via the university’s round mail system and on online social media platforms. At the beginning, the questionnaires for general anxiety, state anxiety, and math anxiety were completed. In the experimental part, participants solved word problems with an overall time limit of 12 min. Further, they were given two example word problems and their correct solutions. Word problems were presented separately and pseudo-randomly (i.e., none of the conditions were presented more than two times in a row). Every correct solution was a two-digit number, so two key presses were required (no correction possible). The first key press indicated response time (RT), while the second key press terminated the trial and led to a fixation cross for 1 s before the next word problem started. Items were centered on the screen with each of the four sentences presented in a new line. After the time limit had expired for the word problems, arithmetic skill for addition and subtraction was assessed. Participants had to type their answer below every problem, and corrections were possible until the time limit of 90 s was over. Finally, participants had 60 s to respond to as many sentences as possible in the reading comprehension test; the next sentence appeared after a given answer until the time limit expired or all sentences were answered. At the end, participants were asked to provide information concerning data quality in web-based studies (Huber & Artemenko, 2021; https://osf.io/3mdb2).

### Design

The study has a 2 × 2 × 2 within-subject design with the factors carry/borrow (with/without), operation (addition/subtraction), and lexical consistency (lexically consistent/inconsistent). Further, math anxiety was added as a continuous variable. General anxiety, test anxiety, reading comprehension ability, and arithmetic ability were considered as further covariates. The main dependent variable for word problem-solving performance was RT; accuracy (number of correctly solved items in relation to the total number of items) was considered in additional analyses.

### Data Analysis

We conducted our data analysis using R (Version 4.4.1, R Core Team, 2021), utilizing several packages including *lme4* (Bates et al., 2015) and *lmerTest* (Kuznetsova et al., 2017) for mixed-effects modeling, *ggplot2* (Wickham et al., 2023) for visualization, and *apaTables* (Stanley, 2024), *psych* (Revelle, 2025) and *misty* (Yanagida, 2025) for handling covariates. All raw data files and complete R analysis scripts are publicly available through an Open Science Framework repository at https://osf.io/tv34c/.

For RT analysis, trials were excluded if incorrectly solved (13.31%), if RTs < 200 ms (0%), and if RT was 3 *SD* above or below the individual *Mean* in an iterative process (20 times; 2.81%). RTs were analyzed using linear mixed-effects models (LMMs) containing the fixed factors operation, carry/borrow, lexical consistency, math anxiety, and their interactions.^4^ We dummy-coded the factors carry (1: with carry/borrow vs. 0: without carry/borrow), operation (1: subtraction vs. 0: addition), and lexical consistency (1: lexically inconsistent form vs. 0: lexically consistent form). In addition, continuous covariates, and the continuous variable math anxiety were grand-mean centered. No missing data were observed for math anxiety due to the forced response format. Accordingly, no data imputation or case exclusion was necessary.

Regarding modeling, we first built the full model with the maximal possible random effect structure, as recommended by Barr et al. (2013). This included random intercepts for participants and items, as well as random slopes for operation, carry/borrow, and lexical consistency, because these factors could cause random variation across participants.^5^ Thus, the full model was

$$\begin{array}{l} y_{ij}=\beta_{0}\;\mathrm{intercept}+\beta_{1}\;\mathrm{math}\;\mathrm{anxiety}+\beta_{2}\mathrm{carry}+\;\beta_{3}\;\mathrm{operation}+\;\beta_{4}\;\mathrm{consistency}+\beta_{5}\;\left(\mathrm{math}\;\mathrm{anxiety}\times \mathrm{carry}\right)+\\ \;\;\;\;\;\;\;\;\;\;\;\;\;\beta_{6}\left(\mathrm{math}\;\mathrm{anxiety}\times \mathrm{operation}\right)+\;\;\beta_{7}\left(\mathrm{math}\;\mathrm{anxiety}\times \mathrm{consistency}\right)+\;\;\beta_{8}\left(\mathrm{carry}\times \mathrm{operation}\right)+\\ \;\;\;\;\;\;\;\;\;\;\;\;\beta_{9}\left(\mathrm{carry}\times \mathrm{consistency}\right)+\;\;\beta_{10}\left(\mathrm{operation}\times \mathrm{consistency}\right)+\beta_{11}\left(\mathrm{math}\;\mathrm{anxiety}\times \mathrm{carry}\times \mathrm{consistency}\right)+\\ \;\;\;\;\;\;\;\;\;\;\;\;\beta_{12}\left(\mathrm{math}\;\mathrm{anxiety}\times \mathrm{carry}\times \mathrm{operation}\right)+\;\;\beta_{13}\left(\mathrm{math}\;\mathrm{anxiety}\times \mathrm{operation}\times \mathrm{consistency}\right)+\\ \;\;\;\;\;\;\;\;\;\;\;\beta_{14}\left(\mathrm{carry}\times \mathrm{operation}\times \mathrm{consistency}\right)+\;\;\beta_{15}\left(\mathrm{math}\;\mathrm{anxiety}\times \mathrm{carry}\times \mathrm{operation}\times \mathrm{consistency}\right)+\\ \;\;\;\;\;\;\;\;\;\;\;v_{0i}+v_{1i}+v_{2i}+v_{3i}+w_{0j}, \end{array}$$

with $v_{0i}\;$ as random intercept for participants, $v_{1i}\;$ as random slope for carry, $v_{2i}\;$ as random slope for operation, $v_{3i}$ as random slope for consistency, all varying across participants, and $w_{0j}\;$ representing the random intercept for items.

After fitting the full LMM with the R-package *lme4* (Bates et al., 2015), both fixed and random effects were tested for significance (with a criterium of *p* < .05) by applying the function *step* from the R-package *lmerTest* (Kuznetsova et al., 2017), which performs automatic backward LMM selection with *F*-tests using Satterthwaite’s method to estimate degrees of freedom. The function tests random effects first and fixed effects in a hierarchical order afterward. Accuracy was analyzed using generalized linear mixed-effects models (GLMM), using the function *drop1* from the R-package *lmerTest* (Kuznetsova et al., 2017), performing backward likelihood ratio tests (i.e., χ^2^ tests). Here, we did not include higher-order interactions or random slopes, because these terms caused convergence issues, suggesting that a simpler model structure better represents the underlying data structure.

Post-hoc power for the highest-order fixed effect of the final LMM (interaction between math anxiety and lexical consistency) was estimated with a Monte Carlo simulation-based sensitivity analysis. Power simulations were performed using the simr package (Green & MacLeod, 2016), which generates datasets by simulating the dependent variable from the fitted model, refitting the model, and testing the target effect across multiple iterations.

## Results

### Word Problem-Solving

The backward selection procedure resulted in the following reduced LMM model for RT (see Table 1):

$$\begin{array}{l} y_{ij}=\beta_{0}\left(\mathrm{intercept}\right)+\beta_{1}\;\mathrm{math}\;\mathrm{anxiety}+\\ \;\;\;\;\;\;\;\;\;\;\;\;\beta_{2}\;\mathrm{carry}+\beta_{4}\;\mathrm{consistency}+\\ \;\;\;\;\;\;\;\;\;\;\;\beta_{7}\left(\mathrm{math}\;\mathrm{anxiety}\times \mathrm{consistency}\right)+\\ \;\;\;\;\;\;\;\;\;\;v_{0i}+v_{1i}+v_{3i}+w_{0j} \end{array}$$

**Table 1.** Response Time – Random and Fixed Effects of the Reduced Model.

Random effects	Variance	*SD*	
Item (Intercept)	1,279,206	1,131	
Subject (Intercept)	4,396,245	2,097	
Subject (Carry)	209,421	458	
Subject (Consistency)	178,479	423	
Residual	8,962,548	2,994	
Fixed effects	Estimate	*SE*	*t*
Intercept	8,278.57	614.95	13.462
Math anxiety	24.21	23.99	1.009
Carry	1,086.923	342.58	3.173
Consistency	78.49	414.75	0.189
Math anxiety × Consistency	28.91	11.11	2.602

**Figure 1.**
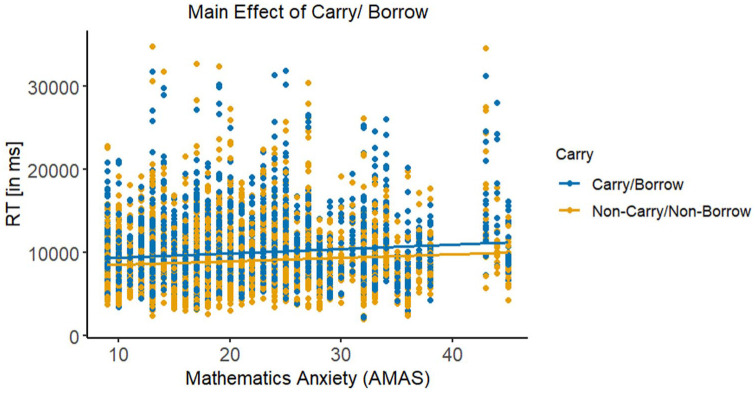
Carry/Borrow effects in word problem-solving. *Note*. The figure shows that the carry or borrow operation generally increased difficulty in word problem-solving. However, this main effect was not further modulated by math anxiety as indicated by AMAS (Abbreviated Math Anxiety Scale) scores.

**Figure 2.**
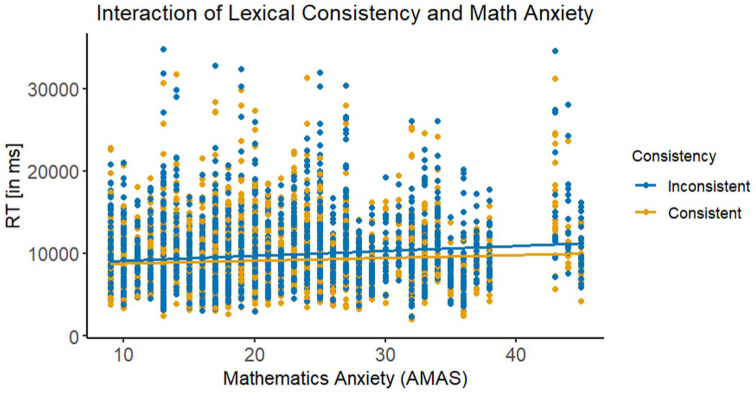
Interaction of lexical consistency and math anxiety in word problem-solving. *Note*. The figure shows response times in word problem-solving as a function of math anxiety as indicated by AMAS (Abbreviated Math Anxiety Scale) scores, grouped by levels of lexical consistency. With increasing self-reported math anxiety, performance was increasingly impaired when facing lexically inconsistent compared to lexically consistent problems.

The reduced model indicates that word problems with a carry or borrow operation need longer to be solved than word problems without these operations (see Figure 1). Moreover, an interaction between math anxiety and consistency was observed: the consistency effect varied as a function of math anxiety, with higher levels of math anxiety being associated with a larger lexical consistency effect (see Figure 2).

The reduced GLMM model for accuracy revealed only a significant main effect of math anxiety (see Table 2), indicating that with increasing math anxiety, performance was less accurate in solving word problems. As overall accuracy was quite high (86.69%), care should be taken in interpreting these results. Consequently, individual differences were also small, as reflected by the comparatively very small individual variation captured by the random effects of participants (see Table 2).

**Table 2.** Accuracy – Random and Fixed Effects of the Reduced Model.

Random effects	Variance	*SD*	
Item (Intercept)	1.3215	1.1496	
Subject (Intercept)	0.3189	0.5647	
Fixed effects	Estimate	*SE*	*Z*
Intercept	2.78	0.25	11.076
Math anxiety	−0.02	0.01	−2.175

Finally, a significant negative correlation between mean RT and mean accuracy across participants, *r*(127) = −.38, *p* < .001, reflected the opposite of a speed-accuracy trade-off, as faster responses were associated with higher accuracy.

The post-hoc test indicated that random slopes should be excluded as some models had fitting issues. The final simulation across 100 iterations yielded approximately 87% power (95% CI [78.80, 92.89]) to detect the observed effect size, suggesting that our study was reasonably well-powered for this interaction.

### Covariates

Table 3 shows the correlations between the covariates, including mean RT, mean accuracy, reading comprehension, and mathematical ability test.

**Table 3.** Means, Standard Deviations, and Correlations With Confidence Intervals.

Variable	*M*	*SD*	1	2	3	4	5	6
1. Word problem response time (RT)	9,547.06	2,405.61						
2. Word problem accuracy (ACC)	0.87	0.08	−.17 [−0.33, 0.00]					
3. Math anxiety (AMAS)	21.22	8.28	.15 [−0.02, 0.32]	−.11 [−0.28, 0.06]				
4. General anxiety (GAD)	7.60	4.31	.11 [−0.06, 0.28]	−.03 [−0.20, 0.14]	.35** [0.19, 0.49]			
5. General state anxiety (STAI)	9.29	3.33	.21* [0.04, 0.37]	−.08 [−0.25, 0.09]	.34** [0.18, 0.49]	.60** [0.47, 0.70]		
6. Reading skill	15.36	6.69	−.35** [−0.49, −0.19]	.01 [−0.16, 0.18]	−.04 [−0.21, 0.14]	.04 [−0.13, 0.21]	−.01 [−0.18, 0.16]	
7. Arithmetic skill	8.69	4.09	−.87** [−0.91, −0.82]	.17 [−0.01, 0.33]	−.24** [−0.39, −0.06]	−.18* [−0.34, −0.01]	−.25** [−0.40, −0.08]	.20* [0.03, 0.36]

*Note. M* and *SD* are used to represent mean and standard deviation, respectively. Values in square brackets indicate the 95% confidence interval for each correlation. The confidence interval is a plausible range of population correlations that could have caused the sample correlation (Cumming, 2014).

AMAS = Abbreviated Math Anxiety Scale; GAD = Generalized Anxiety Disorder; STAI = State and Trait Anxiety Inventory.

* indicates *p* < .05. ** indicates *p* < .01.

In an exploratory analysis, reading comprehension and arithmetic skill were considered in the LMM of word problem-solving (for RT only due to high overall accuracy). Reading comprehension and arithmetic skills did not significantly alter the models, but they improved the model’s fit (see Table 4). Both reading comprehension and arithmetic skills had a significant impact on word problem-solving performance. In addition, the relative magnitudes indicate that arithmetic skill explains most variance in RT, followed by reading skill, both exceeding the contribution of math anxiety. The random effect for subject decreased, indicating that differences in variance between participants can be partially explained by reading and arithmetic skills. Importantly, the interaction between math anxiety and lexical consistency remained in the model. Additionally, main effects of carry/borrow, lexical consistency, and math anxiety were observed.

**Table 4.** Response Time with Reading Skill and Mathematical Skill – Random and Fixed Effects of the Reduced Model.

Random effects	Variance	*SD*	
Item (Intercept)	1,261,925	1,123.4	
Subject (Intercept)	719,305	848.1	
Subject (Carry)	205,289	453.1	
Subject (Consistency)	171,931	414.6	
Residual	8,966,688	2,994.4	
Fixed effects	Estimate	*SE*	*t*
Intercept	8,715.67	300.01	29.05
Math anxiety	−28.89	11.96	−2.42
Carry	1,082.26	340.39	3.18
Consistency	682.15	339.97	2.01
Reading skill	−65.39	14.10	−4.64
Arithmetic skill	−484.80	23.91	−20.28
Math anxiety × Consistency	28.43	11.09	2.57

## Discussion

This study investigated the impact of math anxiety on word problem performance in general and specifically whether math anxiety affects performance based only on arithmetic difficulty (carry/borrow and arithmetic operations) or also on linguistic difficulty (lexical consistency). Our findings reveal that math anxiety impaired performance in word problems. Moreover, math anxiety interacted with linguistic difficulty so that math anxiety particularly impacted word problems when the text was more difficult to translate into an arithmetic problem. On a theoretical level, we conclude that math anxiety particularly impairs solving linguistically difficult word problems, but not necessarily arithmetically difficult word problems.

### Influence of Arithmetic and Linguistic Difficulty in Word Problems

The difficulty of word problems was found to depend on arithmetic and linguistic difficulty. Replicating previous research on word problems (Daroczy et al., 2020; Dresen et al., 2020), carry/borrow effects were found in the current study, indicating that solving word problems involving calculations with carry or borrow operations took longer compared to word problems without these operations in lexically consistent problems. This is in line with a substantial body of research showing slower responses when arithmetic requires place-value computation, such as carrying in addition and borrowing in subtraction (e.g., Artemenko et al., 2018). Contrary to previous research (e.g., Daroczy et al., 2020), however, we did not find evidence that word problem performance varied by the arithmetic operation.

Regarding linguistic difficulty, the lexical consistency effect was observed after controlling for math skills and reading comprehension. Solving word problems takes longer when the language is inconsistent with the arithmetic operation, replicating previous research (Daroczy et al., 2020; Hegarty et al., 1992, 1995). The difficulty might arise due to greater demands on working memory (Daroczy et al., 2015). Moreover, contrary to our expectations, accuracy was not significantly affected by arithmetic or linguistic complexity. This might be due to ceiling effects in our data, as our subjects had a high accuracy rate (almost 90%).

Most notably, the results indicate that arithmetic and linguistic difficulty impact word problem performance. However, the effect of linguistic difficulty was further modulated by math anxiety.

### Mathematics Anxiety Impacts Word Problem Performance

Math anxiety had a general negative effect on word problem-solving performance in adults. This finding is consistent with the existing literature, which shows a negative association between math anxiety and math performance in general (see Dowker et al., 2016, for a review) and specifically in word problem-solving (see Barroso et al., 2021, for a meta-analysis). This corroborates existing literature on word problems not only in primary school students (e.g., Ramirez et al., 2016) but also in adults (e.g., Novak & Tassell, 2017).

Notably, the influence of task difficulty, as revealed in previous studies, adds another layer to this intricate relationship. While math anxiety generally affects accuracy in word problem-solving, the general influence of math anxiety on RT was only observed after controlling for arithmetic skills and reading comprehension. Moreover, this influence of math anxiety varied depending on the linguistic complexity of the word problem.

### Influence of Mathematics Anxiety on Linguistic but not Arithmetic Difficulty in Word Problems

The current study focused on the question whether math anxiety affects word problem performance particularly when arithmetic and/or linguistic difficulty increases. The findings suggest that indeed this impact of math anxiety was observed for linguistic difficulty – but not for arithmetic difficulty.

Arithmetic difficulty was not affected by math anxiety in the context of word problems. First, math anxiety did not differentially affect the arithmetic operations addition and subtraction, replicating a previous finding from basic arithmetic (Huber & Artemenko, 2021). However, math anxiety did also not differentially affect the carry and borrow effects in arithmetic – and this contrasts with findings in basic arithmetic tasks, where math anxiety particularly impaired arithmetic performance when addition needs carrying and subtraction needs borrowing (Ashcraft, 2002; Ashcraft & Kirk, 2001; Chan & Tang, 2020; Faust et al., 1996; Huber & Artemenko, 2021; Hunt et al., 2014; Hunt & Sandhu, 2017). In the context of word problems, the anxiety-complexity effect could not be replicated in this form, suggesting that the carry/borrow operations are difficult per se, independent from the individual math anxiety level. While arithmetic difficulty due to carry and borrow operations slows down performance, the effect is not moderated by math anxiety, indicating that the anxiety-complexity effect does not generalize to carry/borrow operations in word problem contexts.

This finding might be explained by the assessment of math anxiety and by the characteristics of word problems. The questionnaire used for math anxiety (AMAS) is rather generally addressing anxiety toward mathematics. Previously, the anxiety-complexity effect could only be shown if a modified version of this questionnaire (mAMAS) was used (Huber & Artemenko, 2021), that more specifically focuses on anxiety toward arithmetic. Therefore, the assessment of math anxiety in the current study might be less related to the arithmetic difficulty in word problems, but rather to other, more global difficulties that arise from arithmetic word problems, such as lexical consistency – and this was supported by our data.

Linguistic difficulty was affected by math anxiety, as indicated by a larger lexical consistency effect for individuals with higher levels of math anxiety. This suggests that individuals with high math anxiety are more affected by ambiguous or misleading wording in word problems (e.g., keywords suggesting addition when subtraction is required). One possible explanation is that math-anxious individuals tend to allocate more cognitive resources to managing their anxiety. This would leave fewer resources available for processing complex or conflicting linguistic cues, thus increasing the time for solving word problems. It shows that individuals with higher mathematics anxiety might struggle not only with numerical difficulty but also with how mathematical problems are formulated, especially when that wording is not straightforward.

Finally, the consideration of reading comprehension and mathematical skills showed essential interindividual differences. This suggests that individual differences play a crucial role in word problem-solving (e.g., Passolunghi et al., 2019), presenting another avenue for potential investigation in the future. On the other hand, the interaction of math anxiety and lexical consistency remained after controlling for arithmetic skills and reading comprehension. This underlines the robustness of the results and thus the specific impact of math anxiety on word problem performance.

Taken together, word problem-solving performance in adults is influenced by both arithmetic and linguistic difficulty. Moreover, math anxiety affected word problem performance, particularly if the linguistic – but not the arithmetic – difficulty increases. This might show that language-related processing demands may dominate in word problems, potentially overshadowing subtle arithmetic differences. Nevertheless, a kind of anxiety-complexity effect was shown – but for linguistically (and not arithmetically) complex word problems. Hence math-anxious individuals might struggle more with the language complexity, rather than arithmetic complexity, when solving word problems.

### Limitations

Some limitations warrant consideration and might motivate future studies in this field. First, gender differences were found in mathematical cognition and word problem-solving performance (e.g., Zhu, 2007) on the one hand, and in math anxiety (Hart & Ganley, 2019) on the other hand. While women show higher levels of math anxiety and also a tendency to avoid more difficult math tasks (Zohar & Gershikov, 2008), they also show better reading comprehension skills (Lietz, 2006). Therefore, the role of gender in the anxiety-complexity effect targeting at linguistic difficulty in word problems is unclear. In our sample, the gender distribution was not equal, with approximately four times more females than males.

Second, the differentiation between perceived difficulty of mathematics and actual mathematical difficulty is noteworthy (e.g., Doz et al., 2023). It remains uncertain whether participants perceived our tasks as more challenging than they actually are, potentially influencing their anxiety reaction and task performance. For example, the word problems could have been too easy for the highly educated web-based sample of adults that was recruited in the current study, which might have underestimated the impact of math anxiety.

Third, while this study focused primarily on the hypothesis that math anxiety negatively impacts word problem performance, we acknowledge that the reverse direction is also well-supported in the literature (e.g., Carey et al., 2016). Given the cross-sectional nature of our design, we are unable to draw solid conclusions about causality or the directionality of effects. Therefore, our findings are equally compatible with both theoretical accounts.

Fourth, a limitation of the current study is the absence of a working memory measure. Including a valid working memory task would have significantly increased the length of the testing session, which may have affected participant engagement – especially in an online setting. Nonetheless, given the well-documented links between working memory (e.g., Pellizzoni et al., 2022) and mathematics anxiety, future research in more controlled environments should consider incorporating working memory to better understand its role in the context of math anxiety and performance.

Fifth, notably, a subset of participants dropped out at different stages of the experiment. Those who aborted the experiment after the first word problems descriptively showed slightly higher levels of math anxiety than later dropouts or completers, although this difference was not statistically significant (*t*(157) = 1.06, *p* = .289). Nevertheless, we cannot exclude the possibility that even a modest, nonsignificant selection bias may have led to a slight underestimation of the observed anxiety-related effects. However, the lack of significant group differences suggests that any such bias is unlikely to have had a major impact on the results.

Finally, findings from web-based experiments might differ from traditional laboratory experiments due to factors such as sample characteristics, sample size, environmental conditions, experimental control, and RT. For instance, web-based experiments offer a diverse participant pool and larger sample sizes, but participants vary in motivation, potentially introducing a higher drop-out rate (e.g., Dandurand et al., 2008). Web-based experiments also lack the same level of control that lab-based experiments offer. However, we were careful to account for this by using screening questions for data quality and linear mixed models for statistical data analysis.

### Perspectives

First, because we examined only change-type word problems, generalizability to other types of word problems (combine, compare) remains to be tested; future work should implement matched designs across different types of word problems to evaluate whether the observed pattern generalizes beyond change-type word problems.

This study highlights the importance of considering emotional factors in mathematics education, especially as math tasks become more complex in advancing curricula. The findings suggest that students with higher levels of math anxiety may struggle not only with arithmetically complex tasks but also with the linguistic demands of word problems. These insights emphasize the need for interventions that address both cognitive and emotional factors in mathematics education. Therefore, teaching should focus not only on the arithmetic in word problems but also on the linguistic aspects of word problems. For instruction and assessment, it may be helpful to separate linguistics from arithmetic difficulty, for example, by using paired problems with the same numbers but clearer or alternative wording, and to employ adaptive and individualized learning systems that target math-anxious students to explain and simplify linguistically more difficult word problems. In future studies, it would be valuable to investigate teachers’ perceptions of math anxious students’ preferences for math problems presented in different formats (e.g., arithmetic vs. word problems), to better understand whether these perceptions align with the empirical findings reported here.

## Conclusion

This study examined the impact of math anxiety on word problem performance in adults, focusing on how this influence varies with arithmetic (complexity and operations) and linguistic (lexical consistency) difficulty. The results showed that math anxiety affected word problem performance. While the difficulty of word problems increased when carry or borrow operations are necessary, this effect was not moderated by math anxiety. Instead, linguistic difficulty was affected by math anxiety, suggesting that math-anxious individuals rather struggle during word problem-solving when the language in word problems is not consistent to the arithmetic operation that needs to be used. This study highlights the importance of considering emotional factors in mathematics education, especially as math tasks become more complex in advancing curricula. The findings suggest that students with higher levels of math anxiety may struggle not only with arithmetically complex tasks but also with the linguistic demands of word problems. These insights emphasize the need for interventions that address both cognitive and emotional factors in mathematics education, allowing educators to enhance students’ mathematical performance, particularly in tasks that require the integration of math and language skills.

## Supplemental Material

sj-docx-1-qjp-10.1177_17470218261425251 – Supplemental material for The Mathematics Anxiety-Complexity Effect in Word Problems Supplemental material, sj-docx-1-qjp-10.1177_17470218261425251 for The Mathematics Anxiety-Complexity Effect in Word Problems by Gabriella Daroczy, Felix Cramer, Christina Artemenko, Thomas E. Hunt and Hans-Christoph Nuerk in Quarterly Journal of Experimental Psychology

## Notes

1. Due to recruitment problems in the United Kingdom (only *N* = 6), the sample was restricted to German-speaking participants so that the preregistered hypothesis H3 on the generalization across languages (German/English) could not be evaluated.

2. Originally, we preregistered a target of *N* = 200 per language group (English and German; total *N* = 400). Due to testing issues, that is, a low response rate in the English sample and high early drop out in the German sample, the final sample was restricted to one language group and the resulting sample size lower than expected. While this does not allow for between-language comparisons, the available sample is sufficient to detect the anxiety–complexity interaction within one sample.

3. Subjects dropped out at various time points: *n* = 44 left the study after the questionnaires and before the first word problem was presented (AMAS: *M* = 20.43, *SD* = 7.21). Out of *n* = 150 who responded to all word problems, *n* = 120 did not continue with the reading test (AMAS: *M* = 20.46, *SD* = 7.39), and *n* = 30 did not verify the honesty of their answers at the end (AMAS: *M* = 19.5, *SD* = 7.71). Interestingly, *n* = 30 dropped out while working on the first couple of word problems (AMAS: *M* = 23.0, *SD* = 8.12), and *n* = 2 completed the whole experiment without responding to the word problems (AMAS: 27 and 35).

4. The preregistration included additionally an extreme group analysis, but we decided against this analysis because it would reduce statistical power by discarding valuable data.

5. Interaction of slopes were discarded, because they made the model instable due to explaining minimal or no variance at all (see LMM remedies by Brauer & Curtin, 2018).
