# Supplementary material for: The Mathematics Anxiety-Complexity Effect in Word Problems
Source: Q J Exp Psychol (Hove). 2026 Feb 17;79(8):2184–98. doi: 10.1177/17470218261425251 (PMC13400789; doi:10.1177/17470218261425251)
Supplement: sj-docx-1-qjp-10.1177_17470218261425251 – Supplemental material for The Mathematics Anxiety-Complexity Effect in Word Problems [file sj-docx-1-qjp-10.1177_17470218261425251.docx]

**Supplementary Material to the Paper:**

**The mathematics anxiety-complexity effect: the relevance of arithmetic and linguistic complexity on word problem-solving performance**

**Table S1**

*Word Problem Stimuli*

| Block | Nr. | Sentences | Condition | |
| --- | --- | --- | --- | --- |
| B1 | | | | |
|  | 1 | S1: Ein Mann spart Geld für einige Anschaffungen.  *Translation: A man saves money on some purchases.*  S2: Er hatte 21 Euro.  *Translation: He had 21 Euros.*  S3: Er verdient 13 Euro.  *Translation: He earns 34 Euros.*  S4: Wie viel Geld hat der Mann jetzt?  *Translation: How much money does the man have now?* | C A nC | |
|  | 2 | S1: Ein paar Freunde suchen Pilze im Wald.  *Translation: A few friends are looking for mushrooms in the forest.*  S2: Sie sammeln gerade 13 Pilze.  *Translation: They are currently collecting 13 mushrooms.*  S3: Sie hatten 36 Pilze davor.  *Translation: They had 36 mushrooms bevor.*  S4: Wie viele Pilze haben sie am Ende?  *Translation: How many mushrooms do they have in the end?* | C A nC | |
|  | 3 | S1: Eine Studentin muss Wörter lernen.  *Translation: A student must learn words.*  S2: Die Studentin kannte 52 Wörter.  *Translation: The student knew 52 words.*  S3: Sie lernte gerade 26 Wörtern.  *Translation: She just learned 26 words.*  S4: Wie viele Wörter kennt die Studentin am Ende? | C A nC | |
|  |  | *Translation: How many words does the student know in the end?* |  | |
|  | 4 | S1: Ein Mädchen sammelt Bücher.  *Translation: A girl collects books.*  S2: Sie hat 35 Bücher erhalten.  *Translation: She receives 35 books..*  S3: Das Mädchen hatte davor 54 Bücher.  *Translation: The girl had 54 books bevor.*  S4: How many books does she have in the end?  *Translation: How many diamonds does she have now?* | C A nC | |
|  | 5 | S1: Ein Dieb hat einer Frau einige Diamanten gestohlen.  *Translation: A thief stole some diamonds from a woman.*  S2: Nach dem Diebstahl hatte sie 23 Diamanten.  *Translation: After the theft she had 23 diamonds.*  S3: Die Polizei findet 15 Diamanten wieder.  *Translation: The police recover 15 diamonds.*  S4: Wie viele Diamanten hat die Frau am Ende?  *Translation: How many diamonds does the woman have in the end?* | C A nC | |
|  | 6 | S1: Einige Leute wurden zur Party eingeladen.  *Translation: Some people were invited to the party.*  S2: 12 Leute sind später gekommen.  *Translation: 12 people came later.*  S3: Es waren davor 75 Leute auf der Party.  *Translation: There were 75 people at the beginning of the party.* S4: Wie viele Leute waren am Ende auf der Party?  *Translation: How many people were at the party in the end?* | C A nC | |
|  | 7 | S1: Eine Studentin muss Wörter lernen.  *Translation: A student must learn words.*  S2: Die Studentin kannte 43 Wörter.  *Translation: The student knew 43 words.*  S3: Sie hat gerade 19 Wörter gelernt.  *Translation: She just learned 19 words.*  S4: Wie viele Wörter kennt die Studentin am Ende?  *Translation: How many words does the student know in the end?* | C A Ca | |
|  | 8 | S1: Einige Leute wurden zur Party eingeladen.  *Translation: Some people were invited to the party.*  S2: 19 Leute sind später gekommen.  *Translation: 19 people came later.*  S3: Es waren anfangs 24 Leute auf der Party.  *Translation: There were 24 people at the beginning of the party.*  S4: Wie viele Leute waren am Ende auf der Party?  *Translation: How many people were at the party in the end?* | C A Ca | |
|  | 9 | S1: Ein paar Freunde suchen Pilze im Wald.  *Translation: A few friends are looking for mushrooms in the forest.*  S2: Sie hatten 67 Pilze.  *Translation: They had 67 mushrooms.*  S3: Sie sammeln 15 Pilze.  *Translation: They collect 15 mushrooms.*  S4: Wie viele Pilze haben sie am Ende?  *Translation: How many mushrooms do they have in the end?* | C A Ca | |
|  | 10 | S1: Eine Tennisspielerin spielt Turniere.  *Translation: A tennis player plays tournaments.*  S2: Sie hat 16 Spiele in dieser Saison gewonnen.  *Translation: She won 16 games this season.*  S3: Sie hat vor dieser Saison 25 Spiele gewonnen  *Translation: She has won 25 games before this season.*  S4: Wie viele Spiele hat sie in ihrer Karriere gewonnen?  *Translation: How many games has she won in her career?* | C A Ca | |
|  | 11 | S1: Ein Mädchen sammelt Bücher.  *Translation: A girl brought collects books.*  S2: Sie erhält 18 Bücher.  *Translation: She receives 18 books.*  S3: Das Mädchen hatte am Anfang 46 Bücher gehabt.  *Translation: The girl had 46 books in the beginning.*  S4: Wie viele Bücher hat sie am Ende?  *Translation: How many books does she have in the end?* | C A Ca | |
|  | 12 | S1: Eine Marktfrau verkauft Äpfel auf dem Markt  *Translation: A market woman sells apples in the marketplace.*  S2: Die Marktfrau kam mit 56 Äpfeln.  *Translation: The market woman came with 56 apples.*  S3: Sie hat 27 Äpfel geliefert bekommen.  *Translation: She got a delivery of 27 apples.*  S4: Wie viele Äpfel hat sie am Ende?  *Translation: How many apples does she have in the end?* | C A Ca | |
|  | 13 | S1: Eine Marktfrau verkauft Äpfel auf dem Markt.  *Translation: A market woman sells apples in the marketplace.*  S2: Die Marktfrau verkauft 13 Äpfel.  *Translation: The market woman sells 18 apples.*  S3: Sie hatte 25 Äpfel mitgebracht.  *Translation: She brought 34 apples.*  S4: Wie viele Äpfel hat sie am Ende?  *Translation: How many apples does she have in the end?* | C S nC | |
|  | 14 | S1: Eine Studentin muss Wörter lernen.  *Translation: A student must learn words.*  S2: Die Studentin vergisst 12 Wörter.  *Translation: The student forgets 12 words.*  S3: Sie hat am Anfang 58 Wörter gelernt.  *Translation: She learned 58 words in the beginning.*  S4: Wie viele Wörter hat sie richtig gelernt?  *Translation: How many words did she learn correctly?* | C S nC | |
|  | 15 | S1: Ein Mann spart Geld für einige Anschaffungen.  *Translation: A man saves money for some purchases.*  S2: Er hatte 37 Euro gehabt.  *Translation: He had 37 Euros.*  S3: Er hat 14 Euro ausgegeben.  *Translation: He spent 14 Euros.*  S4: Wie viel Geld hat er am Ende?  *Translation: How much money does he have in the end?* | C S nC | |
|  | 16 | S1: Eine Tennisspielerin spielt Turniere.  *Translation: A tennis player plays tournaments.*  S2: Sie hat in dieser Saison 59 Spiele gespielt.  *Translation: She has played 59 games this season.*  S3: Sie hat 41 Spiele verloren.  *Translation: She lost 41 games.*  S4: Wie viele Spiele hat sie gewonnen?  *Translation: How many games did she win?* | C S nC | |
|  | 17 | S1: Ein Mädchen sammelt Bücher.  *Translation: A girl collects books.*  S2: Das Mädchen hat 28 Bücher.  *Translation: The girl has 28 books.*  S3: Sie verschenkt 13 Bücher an ihre Freunde.  *Translation: She gifts 13 books to her friends.*  S4: Wie viele Bücher hat sie am Ende?  *Translation: How many books does she have in the end?* | C S nC |  |
|  | 18 | S1: Ein Dieb hat einer Frau einige Diamanten gestohlen.  *Translation: A thief stole some diamonds from a woman.*  S2: Ihr wurden 32 Diamanten gestohlen.  *Translation: 32 of her diamonds were stolen.*  S3: Die Frau hatte 53 Diamanten gehabt.  *Translation: The woman had 53 diamonds,*  S4: Wie viele Diamanten hat sie jetzt?  *Translation: How many diamonds does she have now?* | C S nC |  |
|  | 19 | S1: Ein Dieb hat einer Frau einige Diamanten gestohlen.  *Translation: A thief stole some diamonds from a woman.*  S2: Der Dieb hat ihr 16 Diamanten gestohlen  *Translation: The thief stole 16 diamonds from her.*  S3: Sie hatte am Anfang 41 Diamanten gehabt.  *Translation: She had 41 diamonds in the beginning.*  S4: Wie viele Diamanten hat sie jetzt?  *Translation: How many diamonds does she have now?* | C S Ca |  |
|  | 20 | S1: Eine Marktfrau verkauft Äpfel auf dem Markt  *Translation: A market woman sells apples in the marketplace.*  S2: Die Marktfrau kam mit 37 Äpfeln.  *Translation: The market woman came with 37 apples.*  S3: Sie verkauft 19 Äpfel.  *Translation: She sells 19 apples.*  S4: Wie viele Äpfel hat sie am Ende?  *Translation: How many apples does she have in the end?* | C S Ca |  |
|  | 21 | S1: Eine Tennisspielerin spielt Turniere.  *Translation: A tennis player plays tournaments.*  S2: Sie verlor 18 Spiele.  *Translation: She lost 18 games.*  S3: Sie hat in dieser Saison 34 Spiele gespielt.  *Translation: She has played 34 games this season.*  S4: Wie viele Spiele hat sie gewonnen?  *Translation: How many games did she win?* | C S Ca |  |
|  | 22 | S1: Ein Mädchen sammelt Bücher.  *Translation: A girl collects books.*  S2: Das Mädchen verschenkt 17 Bücher.  *Translation: The girl gifts 17 books.*  S3: Sie hatte 46 Bücher mitgebracht.  *Translation: She brought 46 books.*  S4: Wie viele Bücher hat sie am Ende?  *Translation: How many books does she have in the end?* | C S Ca |  |
|  | 23 | S1: Einige Leute wurden zur Party eingeladen,  *Translation: Some people were invited to the party.*  S2: 41 Leute sind zur Party gekommen.  *Translation: 41 people came to the party.*  S3: 25 Leute haben die Party früher verlassen.  *Translation: 25 people left the party early.*  S4: Wie viele Leute sind jetzt auf der Party?  *Translation: How many people are at the party now?* | C S Ca |  |
|  | 24 | S1: Ein paar Freunde suchen Pilze im Wald.  *Translation: A few friends are looking for mushrooms in the forest.*  S2: Sie haben 65 Pilze gefunden.  *Translation: They found 65 mushrooms.*  S3: Sie haben 36 giftige Pilze weggeworfen.  *Translation: They throw 36 poisonous mushrooms away.*  S4: Wie viele Pilze haben sie danach?  *Translation: How many mushrooms do they have afterwards?* | C S Ca |  |
|  | 25 | S1: Ein paar Freunde suchen Pilze im Wald.  *Translation: A few friends are looking for mushrooms in the forest.*  S2: Sie werfen gerade 23 giftige Pilze weg.  *Translation: They now throw away 23 poisonous mushrooms.*  S3: Sie haben 31 Pilze übrig.  *Translation: They have 31 mushrooms.*  S4: Wie viele Pilze hatten sie früher?  *Translation: How many mushrooms did they have earlier?* | I A nC |  |
|  | 26 | S1: Eine Marktfrau verkauft Äpfel auf dem Markt.  *Translation: A market woman sells apples in the marketplace.*  S2: Die Marktfrau verkauft 13 Äpfel.  *Translation: She sells 13 apples.*  S3: Sie hat danach 36 Äpfel*.*  *Translation: She has 36 apples afterwards.*  S4: Wie viele Äpfel hatte sie am Anfang?  *Translation: How many apples did she have in the beginning?* | I A nC |  |
|  | 27 | S1: Einige Leute wurden zur Party eingeladen.  *Translation: Some people were invited to the party.*  S2: 72 Leute haben die Party verlassen.  *Translation: 72 people left the party.*  S3: Es blieben danach 26 Leute.  *Translation: There remained 26 people afterwards.*  S4: Wie viele Leute waren zur Party gekommen?  *Translation: How many people had come to the party?* | I A nC |  |
|  | 28 | S1: Eine Tennisspielerin spielt Turniere.  *Translation: A tennis player plays tournaments.*  S2: Sie hatte früher 23 Spiele verloren.  *Translation: She lost 23 games this season.*  S3: Sie hat 14 Spiele in dieser Saison verloren.  *Translation: She had previously lost 14 games.*  S4: Wie viele Spiele hat sie in ihrer Karriere verloren?  *Translation: How many games has she lost in her career?* | I A nC |  |
|  | 29 | S1: Ein Dieb hat einer Frau einige Diamanten gestohlen.  *Translation: A thief stole some diamonds from a woman.*  S2: Sie hat jetzt 34 Diamanten.  *Translation: She now has 34 diamonds*  S3: Der Dieb hatte ihr 25 Diamanten gestohlen.  *Translation: The thief stole 25 diamonds from her.*  S4: Wie viele Diamanten hatte die Frau?  *Translation: How many diamonds did the woman have?* | I A nC |  |
|  | 30 | S1: Ein Mann spart Geld für einige Anschaffungen.  *Translation: A man saves money for some purchases.*  S2: Er hat jetzt 13 Euro.  *Translation: He now has 13 Euros*  S3: Er hat davor 65 Euro ausgegeben.  *Translation: He spent 65 Euros earlier.*  S4: Wie viel Geld hatte der Mann?  *Translation: How much money did the man have?* | I A nC |  |
|  | 31 | S1: Ein Mann spart Geld für einige Anschaffungen.  *Translation: A man saves money for some purchases.*  S2: Er gibt 19 Euro aus.  *Translation: He spends 19 Euros.*  S3: Er hat jetzt 27 Euro.  *Translation: He now has 27 Euros.*  S4: Wie viel Geld hatte der Mann?  *Translation: How much money did the man have?* | I A Ca |  |
|  | 32 | S1: Ein paar Freunde suchen Pilze im Wald.  *Translation: A few friends are looking for mushrooms in the forest.*  S2: Sie werfen gerade 19 giftige Pilze weg.  *Translation: They now throw 19 poisonous mushrooms away.*  S3: Sie haben 45 Pilze.  *Translation: They have 45 mushrooms.*  S4: Wie viele Pilze hatten sie davor?  *Translation: How many mushrooms did they have earlier?* | I A Ca |  |
|  | 33 | S1: Eine Studentin muss Wörter lernen.  *Translation: A student must learn words.*  S2: Die Studentin hat 53 Wörtern vergessen.  *Translation: The student forgot 53 words.*  S3: Sie lernt später 18 andere Wörter.  *Translation: She later learns 18 other words.*  S4: Wie viele Wörter müsste sie kennen?  *Translation: How many words should she know?* | I A Ca |  |
|  | 34 | S1: Ein Mädchen sammelt Bücher.  *Translation: A girl brought her friends books.*  S2: Das Mädchen verschenkt 25 Bücher.  *Translation: The girl gifts 25 books.*  S3: Sie hat danach 17 Bücher.  *Translation: She has 17 books afterwards.*  S4: Wie viele Bücher hatte sie am Anfang?  *Translation: How many books did she have in the beginning?* | I A Ca |  |
|  | 35 | S1: Eine Marktfrau verkauft Äpfel auf dem Markt.  *Translation: A market woman sells apples in the marketplace.*  S2: Sie hat jetzt 38 Äpfel.  *Translation: She now has 38 apples.*  S3: Die Marktfrau hatte davor 26 Äpfel verkauft.  *Translation: The market woman sold 26 apples earlier.*  S4: Wie viele Äpfel hatte sie am Anfang?  *Translation: How many apples did she have in the beginning?* | I A Ca |  |
|  | 36 | S1: Eine Tennisspielerin spielt Turniere.  *Translation: A tennis player plays tournaments.*  S2: Sie hat in dieser Saison 17 Spiele verloren.  *Translation: She has lost 17 games this season.*  S3: Sie hatte früher 76 Spiele verloren.  *Translation: She had previously lost 76 games.*  S4: Wie viele Spiele hat sie in ihrer Karriere verloren?  *Translation: How many games has she lost in her career?* | I A Ca |  |
|  | 37 | S1: Einige Leute wurden zur Party eingeladen.  *Translation: Some people were invited to the party.*  S2: Es sind 25 Leute auf der Party.  *Translation: There are 25 people at the party.*  S3: 12 Leute sind später gekommen.  *Translation: 12 people came later.*  S4: Wie viele Leute waren am Anfang auf der Party?  *Translation: How many people were in the beginning at the party?* | I S nC |  |
|  | 38 | S1: Ein Dieb hat einer Frau einige Diamanten gestohlen.  *Translation: A thief stole some diamonds from a woman.*  S2: Die Polizei hat 13 Diamanten wiedergefunden.  *Translation: The police recovered 13 diamonds.*  S3: Der Dieb hatte ihr 37 Diamanten gestohlen.  *Translation: The thief had stolen 37 diamonds from her.*  S4: Wie viele Diamanten muss die Polizei noch finden?  *Translation: How many diamonds are left for the police to find?* | I S nC |  |
|  | 39 | S1: Eine Tennisspielerin spielt Turniere.  *Translation: A tennis player plays tournaments in singles.*  S2: Sie hat in dieser Saison 29 Spiele gespielt.  *Translation: She has played 29 games this season.*  S3: Sie gewann 13 Spiele.  *Translation: She won 13 games.*  S4: Wie viele Spiele hatte sie verloren?  *Translation: How many games had she lost?* | I S nC |  |
|  | 40 | S1: Eine Studentin muss Wörter lernen.  *Translation: A student must learn words.*  S2: Die Studentin hat 42 Wörter gelernt.  *Translation: The student learned 42 words.*  S3: Sie kennt jetzt 68 Wörter.  *Translation: She now knows 68 words.*  S4: Wie viele Wörter hatte sie am Anfang gekonnt?  *Translation: How many words did she know in the beginning?* | I S nC |  |
|  | 41 | S1: Eine Marktfrau verkauft Äpfel auf dem Markt.  *Translation: A market woman sells apples in the marketplace.*  S2: Sie hatte vorher 21 Äpfel geliefert bekommen.  *Translation: She got a delivery of 21 apples before.*  S3: Die Marktfrau hat 64 Äpfel.  *Translation: The market woman has 64 apples.*  S4: Wie viele Äpfel hatte sie am Anfang?  *Translation: How many apples did she have in the beginning?* | I S nC |  |
|  | 42 | S1: Ein Mann spart Geld für einige Anschaffungen.  *Translation: A man saves money for some purchases.*  S2: Er hat jetzt 37 Euro.  *Translation: He now has 37 Euros.*  S3: Er hatte 24 Euro verdient.  *Translation: He had earned 24 Euros.*  S4: Wie viel Geld hatte er am Anfang?  *Translation: How much money did he have in the beginning?* | I S nC |  |
|  | 43 | S1: Ein paar Freunde suchen Pilze im Wald.  *Translation: A few friends are looking for mushrooms in the forest.*  S2: Sie haben am Ende 31 Pilze.  *Translation: They have 31 mushrooms in the end.*  S3: Sie haben davor 12 Pilze gesammelt.  *Translation: They collected 12 mushrooms earlier.*  S4: Wie viele Pilze hatten sie am Anfang?  *Translation: How many mushrooms did they have in the beginning?* | I S Ca |  |
|  | 44 | S1: Ein Mann spart Geld für einige Anschaffungen.  *Translation: A man saves money for some purchases.*  S2: Er verdient heute 19 Euro.  *Translation: He earns today 19 Euros.*  S3: Er hat jetzt 37 Euro.  *Translation: He now has 37 Euros.*  S4: Wie viel Geld hatte er am Anfang?  *Translation: How much money did he have in the beginning?* | I S Ca |  |
|  | 45 | S1: Eine Studentin muss Wörter lernen.  *Translation: A student must learn words.*  S2: Die Studentin hat 13 Wörter gelernt.  *Translation: The student learned 13 words.*  S3: Sie kennt jetzt 31 Wörter.  *Translation: She now knows 31 words.*  S4: Wie viele Wörter hatte sie am Anfang gekonnt?  *Translation: How many words did she know in the beginning?* | I S Ca |  |
|  | 46 | S1: Ein Dieb hat einer Frau einige Diamanten gestohlen.  *Translation: A thief stole some diamonds from a woman.*  S2: Der Dieb hatte ihr 54 Diamanten gestohlen.  *Translation: The thief had stolen 54 diamonds from her.*  S3: Die Polizei findet 28 Diamanten wieder.  *Translation: The police recover 28 diamonds.*  S4: Wie viele Diamanten muss die Polizei noch finden?  *Translation: How many diamonds are left for the police to find?* | I S Ca |  |
|  | 47 | S1: Ein Mädchen sammelt Bücher.  *Translation: A girl brought her friends books.*  S2: Das Mädchen erhält 45 Bücher.  *Translation: The girl receives 45 books*  S3: Sie hat danach 74 Bücher.  *Translation: She has 74 books afterwards.*  S4: Wie viele Bücher hatte sie am Anfang?  *Translation: How many books did she have in the beginning?* | I S Ca |  |
|  | 48 | S1: Einige Leute wurden zur Party eingeladen.  *Translation: Some people were invited to the party.*  S2: Am Ende sind 41 Leute auf der Party.  *Translation: In the end there are 41 people at the party.*  S3: 18 Leute sind davor zur Party gekommen.  *Translation: 18 people came to the party earlier.*  S4: Wie viele Leute waren am Anfang auf der Party?  *Translation: How many people were there at the beginning of the party?* | I S Ca |  |

S1: First sentence. S2: Second sentence. S3: Third sentence. S4: Fourth Sentence. C: Consistent Form. I: Inconsistent Form. S: Subtraction. A: Addition. Ca: Carry/Borrow. nC: Non-Carry/Non-Borrow
